# Tislelizumab-induced psoriasis in a patient with hepatocellular carcinoma: A case report

**DOI:** 10.3892/etm.2026.13314

**Published:** 2026-09-23

**Authors:** Zhaochen Sun, Yuqi Zhou, Yeliang Mei, Yitian Chen

**Affiliations:** 1 Department of Medical Oncology, Jinling Hospital, School of Medicine, Nanjing University, Nanjing, Jiangsu 210018, P.R. China; 2 Department of Hematology and Oncology, 904 Hospital of People's Liberation Army Joint Logistic Support Force, Wuxi, Jiangsu 214000, P.R. China; 3 Department of Dermatology and Venereology, 904 Hospital of PLA Joint Logistic Support Force, Wuxi, Jiangsu 214000, P.R. China

**Keywords:** tislelizumab, psoriasis, immune-related adverse events, programmed cell death protein 1 inhibitor, narrow-band ultraviolet B irradiation, hepatocellular carcinoma

## Abstract

Immune checkpoint inhibitors (ICIs) are widely incorporated into first-line treatment regimens for advanced hepatocellular carcinoma (HCC). Nevertheless, the growing incidence of immune-related adverse events (irAEs), particularly dermatologic toxicities, poses a substantial challenge to the continuation of anticancer therapy. The present study describes a case of tislelizumab-associated psoriasis in a patient with hepatitis C virus (HCV)-related HCC, emphasizing the need for prompt recognition and individualized management. A 60-year-old man with stage IIIA HCC and chronic HCV infection developed progressively worsening erythematous plaques with scaling during the eighth cycle of tislelizumab plus bevacizumab. Histopathological examination of a skin biopsy supported a diagnosis of pustular psoriasis. A Naranjo Adverse Drug Reaction Probability Scale score of 7 suggested that tislelizumab was the probable causative agent. Because the patient had type 2 diabetes mellitus and declined systemic glucocorticoid treatment, tislelizumab was discontinued, and narrow-band ultraviolet B (NB-UVB) phototherapy was administered in combination with topical corticosteroids. The skin lesions markedly resolved with no recurrence during the 6-week follow-up period. To the best of our knowledge, this is the first report of NB-UVB phototherapy successfully treating ICI-induced psoriasis. This case underscores the need for close monitoring of cutaneous irAEs during ICI therapy and suggests that NB-UVB phototherapy may be an effective alternative to systemic corticosteroids for managing ICI-induced psoriasis, particularly in patients where preservation of antitumor immunity is desirable or systemic steroids are contraindicated. Regular multidisciplinary follow-up and individualized management of adverse events are essential for optimizing both oncological and dermatological outcomes.

## Introduction

Tislelizumab is an innovative immunotherapy comprising a humanized recombinant monoclonal antibody that binds to programmed cell death protein 1 (PD-1) with high specificity. By preventing PD-1 from interacting with its ligands, this immune checkpoint inhibitor (ICI) alleviates tumor-induced T-cell inhibition and reactivates antitumor immunity (1). Introduced to clinical practice in 2019, tislelizumab has become a regular treatment component for various types of cancer, such as lung cancer, hepatocellular carcinoma (HCC) and gastric cancer (2,3). One of the sources of evidence regarding the utility of tislelizumab for HCC is the results of the phase III clinical trial RATIONALE-301, where monotherapy with tislelizumab was proven to be non-inferior in terms of overall survival compared with sorafenib in patients with unresectable HCC. In addition, tislelizumab was associated with a better safety profile and a higher rate of objective responses (4).

As the clinical application of ICIs continues to expand, immune-related adverse events (irAEs) are being identified with increasing frequency (5). Among dermatologic irAEs, psoriasis is a relatively common manifestation and is characterized by a chronic, relapsing inflammatory course (6). Thus far, only one case of psoriasis related to tislelizumab has been documented, to the best of our knowledge (7). In that previous report, management consisted of oral prednisone (0.5 mg/kg/day) together with topical halometasone; however, systemic glucocorticoid administration may be unsuitable for individuals with certain comorbidities, including inadequately controlled diabetes mellitus. Such treatment may also weaken antitumor immune activity (8). Narrow-band ultraviolet B (NB-UVB) phototherapy is an established evidence-based intervention for moderate-to-severe psoriasis. Because its immunomodulatory effects are largely confined to the skin and do not result in extensive systemic immunosuppression (9,10), it may offer particular advantages for patients undergoing cancer treatment.

The present study describes a case of tislelizumab-induced psoriasis in a patient with hepatitis C virus (HCV)-associated HCC. To the best of our knowledge, this is the first reported use of NB-UVB phototherapy for ICI-related psoriasis. This case may inform treatment selection, particularly for patients in whom systemic corticosteroids are contraindicated or undesirable.

## Case report

In January 2025, a 60-year-old man was admitted to 904 Hospital of the People's Liberation Army Joint Logistic Support Force (Wuxi, China) because of pain in the hepatic region. Contrast-enhanced computed tomography of the upper abdomen identified a low-density lesion in the right lobe of the liver. Serum tumor marker testing showed an α-fetoprotein level of 262.09 ng/ml (normal range, <20 ng/ml). Infectious disease screening was positive for HCV antibodies. Subsequent quantitative viral RNA testing showed an HCV RNA level of 2.93x10^6^ IU/ml, indicating active viral replication. Liver biopsy was performed after admission of the patient and histopathological evaluation showed HCC in clinical stage IIIA. There was an established history of smoking for the past 40 years, which the patient quit after starting treatment. Another notable finding was that the patient had type 2 diabetes mellitus (T2DM), which was treated with insulin aspart (30 units, subcutaneous) for glycemic control.

At the initial assessment, the body mass index of the patient was 18.23 kg/m^2^ (slightly below the normal range, indicating underweight status by WHO criteria). Nutritional Risk Screening 2002 showed a score of 1, which implied absence of nutritional risk, whereas the Eastern Cooperative Oncology Group performance status was grade 1 (ambulatory, capable of self-care but unable to perform strenuous activity) (11,12). Between March and July 2025 was marked by eight cycles of 21-day (3-week) cycles of combined treatment with tislelizumab and bevacizumab. During each cycle, 200 mg tislelizumab (intravenous infusion) was applied on the first day along with 500 mg bevacizumab (intravenous infusion). Furthermore, upon beginning treatment, the patient was treated with sofosbuvir 400 mg/velpatasvir 100 mg orally once daily for HCV infection for a period of 3 months. Bicyclol (25 mg orally three times daily) was used for hepatoprotection, and human albumin (10-20 g intravenous infusion) and thymosin α-1 (1.6 mg subcutaneous injection, twice weekly) were supplemented for supportive care. HCV RNA became undetectable in the second month of antiviral treatment.

Administration of the eighth cycle of tislelizumab plus bevacizumab was completed in July 2025, and ~3 days later, the patient exhibited a well-defined, unilateral rash that affected the lumbar and dorsal areas of their body, and was associated with desquamation and pruritus. The rash was considered to be localized eczema, and therefore calamine lotion along with desonide 0.05% cream was prescribed to the patient in addition to continuing the ongoing treatment. However, there was no notable improvement observed in their condition, and the rash worsened and became widespread over ~2 weeks. The patient was admitted in August 2025 for further evaluation and treatment.

On presentation, the patient was conscious and oriented, and the body temperature and blood pressure of the patient were within the normal range. Examination of the skin showed the presence of a number of irregular-shaped red plaques of different sizes scattered all over the body. These lesions had well-defined borders with scaling seen in some of the plaques. There was coalescence of adjacent lesions to form large pustular plaques with positive Auspitz sign (Fig. 1A-C). Notably, >30% of the total body surface area was affected and intense pruritus caused substantial interference with the day-to-day activities of the patient. Based on Common Terminology Criteria for Adverse Events (CTCAE) criteria (8), the severity of the described dermatological side effect was evaluated as grade 3. While the patient had strong pruritus, the lesions were not painful. Histological analysis (skin biopsy samples were fixed in 4% paraformaldehyde for 24-48 h at room temperature, embedded in paraffin, and sectioned at 4-µm thickness. Hematoxylin staining was performed for 3-5 min and eosin for 1-2 min, both at room temperature. Slides were dehydrated in graded ethanol, cleared in xylene, and mounted with neutral balsam. Histopathological examination was performed under a light microscope at x40 magnification) of biopsied lesions showed mild hyperplasia of the central squamous epithelium, neutrophils in the upper dermis, vascular congestion in the dermis and accumulation of collagen fibers (Fig. 1D). The combined clinical and pathological features supported a diagnosis of psoriasis.

After reviewing the treatment timeline and applying the Naranjo Adverse Drug Reaction Probability Scale (13), tislelizumab was judged to be the likely cause of psoriasis. The final causality score was 7 (Table I), corresponding to a probable drug-induced reaction. Although current recommendations for ICI-associated toxicity generally support corticosteroid administration, this option was not pursued. Given the patient's pre-existing type 2 diabetes mellitus and his refusal of both oral and intravenous glucocorticoid therapy, systemic corticosteroid treatment was not pursued.

Following consultation with the dermatology department, treatment with 311-nm NB-UVB irradiation was initiated. Phototesting established a minimal erythema dose of 500 mJ/cm^2^; accordingly, the first session was delivered at 70% of this value, corresponding to 350 mJ/cm². Provided that no phototoxic response developed in the affected skin, the exposure dose was raised by 15% at each subsequent treatment session. Protective eyewear was used, and the genital region was covered throughout treatment. Phototherapy was administered every other day. Beginning in August 2025, the patient received 18 NB-UVB sessions over ~5 weeks. Clinical improvement was first observed after approximately six sessions with marked clearance by week 4.

NB-UVB therapy was considered appropriate for several reasons. It is an established therapeutic modality for extensive moderate-to-severe plaque psoriasis (9) and eliminates the need for systemic glucocorticoids, thereby reducing the likelihood of treatment-related hyperglycemia in individuals with diabetes. Its immunoregulatory activity is largely confined to the skin (10), limiting generalized immune suppression that might otherwise interfere with anticancer immunity. In addition, its overall tolerability makes it suitable for patients with multiple concurrent medical conditions (9).

Topical preparation with 0.05% halometasone and 1% triclosan was used as an adjuvant therapy during the period of phototherapy sessions. The ointment was applied in a thin layer on all lesions twice a day (in the morning and at night) with exception of the face and intertriginous areas. This therapy lasted for 4 weeks with NB-UVB light therapy. To minimize the possibility of relapse after intensive topical therapy, the ointment was applied once a day for 1 week and then alternatively for another week. Thus, the total period of topical therapy was ~6 weeks. There were no side effects such as skin atrophy or telangiectasia.

Regarding antitumor treatment, the patient had three courses of bevacizumab monotherapy between August and November 2025, as well as transcatheter arterial chemoembolization in September 2025, with no adverse effects of the treatment being noted. At re-admission for anticancer therapy in November 2025, revealed that the skin lesions on the trunk had greatly improved compared with previous visits (Fig. 2A-C). No recurrence of psoriasis was observed during the ~6 weeks after discontinuation of NB-UVB phototherapy and topical corticosteroid treatment. Extended follow-up through November 2025, ~10 weeks post-treatment completion, confirmed sustained remission without recurrence. Tislelizumab therapy was not resumed.

## Discussion

The patient described in the present study had no history of psoriasis or known food or drug allergies. A review of their medication history revealed long-term subcutaneous administration of insulin aspart (30 units daily, subcutaneous injection) for glycemic control. Several lines of evidence argue against an association between insulin therapy and the development of psoriasis in this patient. First, the patient had received insulin aspart for several years before rash onset without experiencing any cutaneous adverse reactions. Drug-induced onset typically develop within weeks to months after initiation of a new medication, rather than after years of stable use. Second, insulin-associated cutaneous reactions are generally confined to injection sites and manifest as lipodystrophy, localized erythema or injection-site hypersensitivity, rather than generalized pustular psoriasis (14,15). Third, insulin is a metabolic hormone with no known immunostimulatory effect capable of triggering *de novo* psoriasis through T helper (Th)1/Th17 pathways. Finally, the patient continued insulin therapy without any change in regimen throughout the onset, progression and resolution of psoriasis. If insulin had been the causative agent, continued exposure would have been expected to perpetuate the condition. The absence of disease persistence despite uninterrupted insulin treatment further argues against a causal association.

Before the onset of psoriasis, the patient had received combination therapy with tislelizumab and bevacizumab, together with sofosbuvir/velpatasvir for HCV infection. Bevacizumab has demonstrated favorable efficacy and safety in patients with primary liver cancer (16). Its major adverse events include gastrointestinal perforation, bleeding, hypertension and proteinuria, none of which corresponded to the clinical presentation of the present patient. Moreover, the patient continued bevacizumab monotherapy after discontinuation of tislelizumab without worsening of the existing lesions or development of new lesions, further arguing against bevacizumab as the causative agent. The main adverse effects of sofosbuvir/velpatasvir include fatigue, anemia, nausea and headache (17). The patient completed the full 12-week antiviral regimen without other reported discomfort, making an association between this medication and the rash less likely.

The patient developed rash and pruritus during the eighth cycle of tislelizumab treatment. Previous research has shown that the median time to onset of *de novo* ICI-induced psoriasis is 9.9 treatment cycles (18), consistent with the timing observed in the current case. A recent large-scale systematic review involving 15,340 patients further reported that psoriasiform eruptions account for 3-5% of cutaneous irAEs, with PD-1 inhibitors being the most frequently implicated drug class. The onset of these reactions varies widely, ranging from several weeks to several months after treatment initiation (19). These findings support a plausible temporal relationship between tislelizumab administration and the adverse reaction. According to the Naranjo criteria (20), the causality assessment yielded a score of 7, indicating a probable relationship between tislelizumab and the adverse reaction.

However, the potential contribution of immune reconstitution following successful antiviral treatment for HCV should be acknowledged as a possible confounding factor. Chronic HCV infection causes persistent immune dysregulation, including increased levels of FoxP3-positive regulatory T cells (Tregs) as a mechanism of viral immune evasion (21). Studies have suggested that normalization of serum HCV RNA levels after antiviral therapy may promote the conversion of FoxP3-positive Tregs into IL-17-producing T cells, thereby exacerbating psoriatic lesions (21,22). This immune reconstitution process could theoretically contribute to psoriasis either independently or synergistically with tislelizumab. Because HCV RNA clearance preceded psoriasis onset by several treatment cycles, a synergistic mechanism in which both HCV clearance and PD-1 blockade promoted Th1/Th17 polarization is plausible. This potential confounding factor contributed to the Naranjo score of 7, indicating a probable rather than definite causal relationship. Nevertheless, several findings support tislelizumab as the primary trigger: The timing was consistent with the typical onset of ICI-induced psoriasis, pustular psoriasis is a recognized class effect of PD-1 inhibitors and tislelizumab-induced psoriasis has also been reported in patients without HCV infection.

ICIs have provided novel therapeutic options for oncology; however, the use of ICIs is accompanied by irAEs. The occurrence of these adverse effects occurs in over one-third of patients receiving ICIs (5), and involves the skin, gastrointestinal system, lungs, endocrine glands and the hematopoietic system. Skin manifestations belong to the most frequent irAEs that may affect ≤40% of patients (5). Their clinical presentation ranges from relatively mild or moderate manifestations, including maculopapular, psoriasiform and lichenoid lesions, to uncommon but severe conditions, such as toxic epidermal necrolysis, drug reaction with eosinophilia and systemic symptoms, and Stevens-Johnson syndrome (23). In a retrospective study of ICI-related mucocutaneous toxicity, psoriasiform lesions accounted for ~12% of cases (24). Moreover, accumulating clinical evidence has suggested that several commonly prescribed ICIs may precipitate *de novo* psoriasis or aggravate previously diagnosed disease (25-28).

The biological basis of psoriasis associated with PD-1 inhibition has not been fully elucidated, but excessive immune stimulation and loss of cutaneous immune equilibrium are thought to be central to its development (23,29,30). Several immune cell subsets participate in this process, including neutrophils, dendritic cells, Tregs, and Th1 and Th17 lymphocytes. Of these, the Th1/Th17 axis appears to have a dominant role in psoriatic inflammation (29,31). By releasing Th cells from PD-1-dependent inhibitory signaling, ICIs may enhance the activity of psoriasis-related Th1 and Th17 populations. The resulting imbalance in inflammatory mediators, particularly IL-1, IL-6, IL-17 and IL-22, promotes immune cell activation within the skin and may initiate new lesions or worsen existing psoriasis (26,30,32). T-cell plasticity may also contribute to this process. Elevated levels of FoxP3-positive Tregs have been observed in patients with chronic HCV (21). The immune reconstitution that follows successful antiviral treatment may be relevant to the clinical course observed in the present patient, although this possibility requires further experimental validation.

Psoriasis is not unique to tislelizumab as an irAE and has been reported with nearly all classes of ICIs; however, its incidence differs among ICI classes. PD-1 inhibitors are consistently associated with higher rates of cutaneous irAEs, including psoriasiform lesions, than programmed death-ligand 1 (PD-L1) inhibitors, possibly because PD-1 blockade produces more extensive immune activation than PD-L1 blockade (19). Compared with PD-1 pathway inhibitors, cytotoxic T-lymphocyte-associated protein-4 blockade produces a distinct pattern of dermatologic toxicity and appears to be less commonly associated with psoriasis. There appears to be a higher incidence of cutaneous irAEs and the occurrence of these events is usually more premature if several checkpoint inhibitors are given simultaneously compared to being given individually (19). Tislelizumab has been designed to prevent its binding to Fcγ receptors in such a way that reduces the clearance of T cells from the body via macrophages; thus, it is speculated that it may have an effect on the type or incidence of immune toxicity (4). However, according to the current clinical trial data, the safety profile of tislelizumab does not seem to differ greatly from other PD-1 inhibitors (4). Although the incidence may vary among individual agents, the general principles of diagnosis and management are similar across the PD-1 inhibitor class.

According to the CTCAE, the management of immune-related cutaneous adverse events induced by ICIs such as tislelizumab is guided by toxicity grade (8,33). Grade 1 reactions are generally mild, and ICI therapy may be continued with appropriate symptomatic treatment. For grade 2 or higher reactions, temporary interruption or permanent discontinuation of ICI therapy should be considered, together with systemic treatment using glucocorticoids, intravenous immunoglobulin or plasma exchange when indicated. Additional immunosuppressive therapy may be considered for refractory cutaneous irAEs. Except in patients with severe, potentially fatal cutaneous reactions, ICI therapy may be resumed after symptoms improve to grade 1, following a comprehensive assessment of tumor status and treatment risks.

Conventional treatment of severe ICI-induced psoriasis generally includes high-dose systemic corticosteroids; however, systemic corticosteroid therapy was considered inappropriate in the present case for several reasons. First, the patient had long-standing T2DM requiring insulin therapy. Systemic corticosteroids can cause substantial hyperglycemia by increasing hepatic gluconeogenesis and peripheral insulin resistance, potentially leading to diabetic ketoacidosis or other metabolic complications (8). Second, the patient declined systemic corticosteroid therapy after a detailed discussion of its potential risks and benefits. Third, broad systemic immunosuppression induced by high-dose corticosteroids may theoretically reduce the antitumor efficacy of ICIs, although the available evidence remains inconclusive (5). Therefore, after discontinuation of tislelizumab, an individualized treatment strategy combining NB-UVB phototherapy with topical corticosteroids was adopted.

To the best of our knowledge, the present study is the first reported case of NB-UVB phototherapy successfully used to treat psoriasiform rash induced by an ICI. This represents a clinically important contribution, as the recent large-scale systematic review by Potekaev *et al* (19) explicitly identified phototherapy as an understudied steroid-sparing approach for ICI-related cutaneous adverse events. NB-UVB phototherapy is an established treatment for widespread plaque psoriasis because of its favorable efficacy, convenience and safety profile (9). Cutaneous NB-UVB irradiation induces a series of photochemical and photobiological responses, including depletion of epidermal Langerhans cells, induction of lymphocyte apoptosis, reduction of local cutaneous lymphocyte infiltration and suppression of interferon-γ-mediated immune responses, thereby exerting local immunomodulatory effects (10). High-dose NB-UVB irradiation has also been shown to suppress proinflammatory cytokine expression while increasing anti-inflammatory cytokine expression, thereby reducing or eliminating psoriatic plaques (34,35). Unlike systemic corticosteroids, NB-UVB acts predominantly within the skin and does not cause broad systemic immunosuppression, which may be advantageous in patients with cancer when preservation of antitumor immunity is desirable. In the current case, the therapy successfully treated the skin lesions without causing any notable pain from the treatment. This suggests that NB-UVB phototherapy could be a promising treatment modality in ICI-induced psoriasis, especially in patients who are not suitable for systemic corticosteroid treatment.

Several limitations of the present case report should be acknowledged. First, as this report describes a single patient, the generalizability of the findings is limited. Second, the potential contribution of immune reconstitution following successful HCV treatment represents a confounding factor that cannot be definitively excluded, as reflected by the Naranjo score of 7, indicating a probable rather than definite causal relationship. Third, although the follow-up period was sufficient to document the initial treatment response, is was relatively short, and longer-term follow-up is needed to assess the durability of remission. Fourth, tislelizumab was not reintroduced; therefore, recurrence of psoriasis after rechallenge could not be evaluated, which would have provided additional evidence supporting causality. Finally, because the patient did not resume ICI therapy, whether NB-UVB phototherapy could permit continued ICI treatment remains unclear and warrants future study.

In conclusion, although ICIs have improved treatment opportunities for a number of patients with solid malignancies, associated skin reactions may markedly reduce quality of life, interrupt or postpone anticancer therapy, and occasionally progress to life-threatening disease. Psoriasis attributable to tislelizumab has rarely been documented (7,19). In the present case, pustular psoriasis caused by tislelizumab has shown positive results with NB-UVB light therapy together with topical steroids. This case carries several notable clinical implications: First, it represents the first report of NB-UVB therapy for ICI-related psoriasis, to the best of our knowledge, offering an effective steroid-sparing alternative for patients in whom systemic corticosteroids are contraindicated, thereby filling a recognized knowledge gap in ICI dermatologic toxicity management. Second, the concurrent HCV infection and subsequent immune reconstitution add unique mechanistic complexity, highlighting the need to consider viral hepatitis status as a potential modifier of irAE risk. Third, the localized immunomodulatory mechanism of NB-UVB offers a theoretical advantage in preserving systemic antitumor immunity compared with broad corticosteroid therapy. Such an approach could be used as a steroid-sparing option for patients who cannot be treated using systemic glucocorticoid treatment. Continuous monitoring on behalf of different specialists is necessary to identify both skin and system toxicities during ICI treatment. Treatment strategies should be based on CTCAE classification, as well as taking into account additional diseases, the severity of skin lesion and patient preferences. Increased clinician awareness, careful follow-up and individualized intervention are required to preserve antitumor benefit without compromising patient safety. Additional research should investigate the association between immune restoration and irAE development, and determine the efficacy and safety of NB-UVB phototherapy for ICI-associated cutaneous reactions.

## Figures and Tables

**Figure 1:**
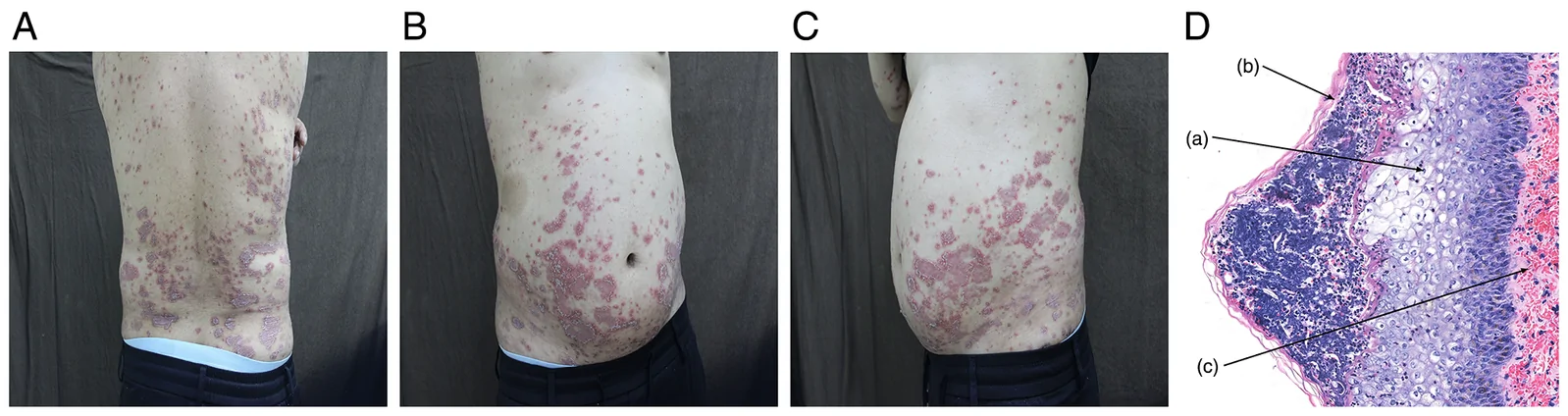
(A) Posterior view of the trunk, showing widespread erythematous plaques and papules with fine scaling involving the lower back, flanks and gluteal region. (B) Anterior view of the trunk, demonstrating generalized psoriatic plaques distributed over the chest and abdomen, with periumbilical involvement. (C) Lateral view of the trunk, showing extensive, confluent erythematous plaques along the lateral chest and abdominal wall. (D) Histopathological examination of the skin biopsy showed mild thickening of the central squamous epithelial layer, superficial neutrophilic infiltration, dermal hyperemia and collagen fiber proliferation (hematoxylin and eosin staining; magnification, x40). (a) Mild squamous epithelial hyperplasia; (b) Sperficial dermal neutrophilic infiltration; and (c) Dermal hyperemia and collagen fiber proliferation.

**Figure 2:**
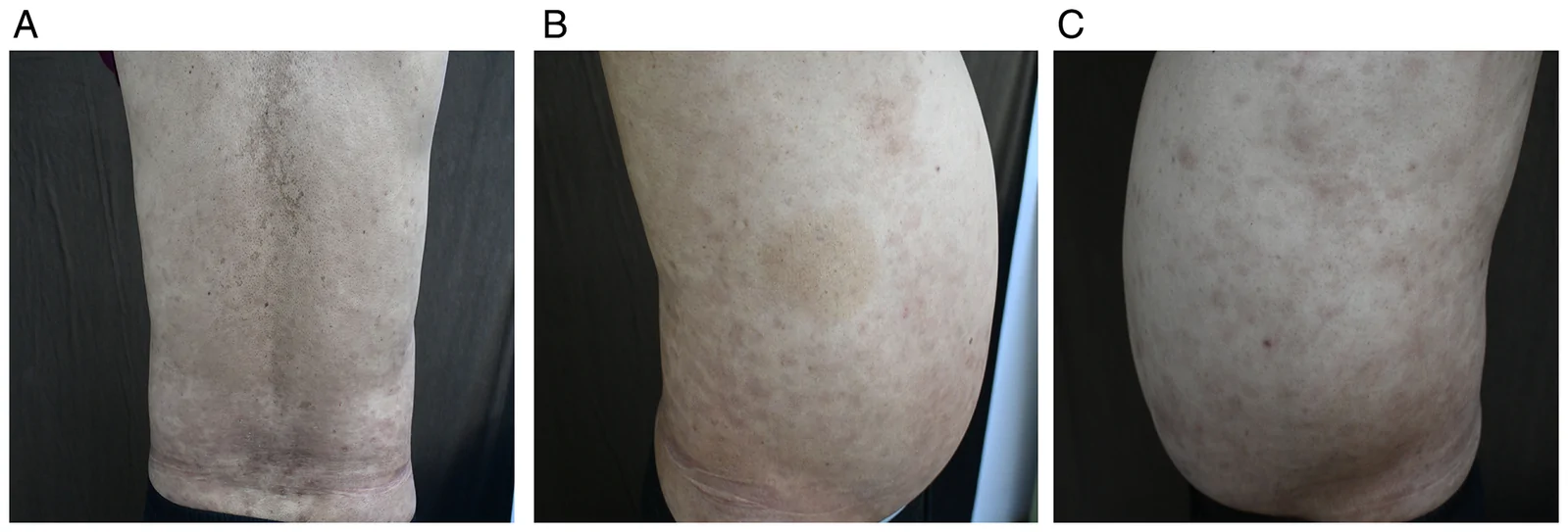
Clinical improvement of psoriatic lesions after NB-UVB phototherapy combined with topical corticosteroid treatment. (A) Posterior view of the trunk after treatment, showing near-complete resolution of psoriatic plaques with residual post-inflammatory hyperpigmentation on the lower back. (B) Anterolateral view of the trunk, demonstrating marked improvement with only scattered residual erythematous macules and post-inflammatory hyperpigmentation. (C) Anterior view of the lower trunk and hip region, showing almost complete clearance of skin lesions with minimal residual pigmentary changes.

**Table I:** Naranjo Adverse Drug Reaction Probability Scale for tislelizumab.

	Question score, points				
Questions	Yes	No	Unknown	Score points	Reasons for rating
1. Has a conclusive report been issued regarding this adverse reaction?	+1	0	0	1	Similar adverse reactions have been documented in the literature, and the official medication package insert also includes reports of rash
2. Did this adverse reaction occur after using the suspected medication?	+2	-1	0	2	This adverse reaction occurred after the use of tislelizumab and is temporally associated with the treatment
3. Did the adverse reaction resolve after discontinuing the medication or administering an antagonist?	+1	0	0	1	Improvement following topical corticosteroid therapy and NB-UVB treatment
4. Did the adverse reaction recur after re-exposure to the suspected medication?	+2	-1	0	0	After the rash improved, tislelizumab was not re-administered
5. Are there other factors that could independently cause this adverse reaction?	-1	+2	0	2	Potential contribution from HCV immune reconstitution cannot be definitively excluded
6. Did this adverse reaction recur after placebo administration?	-1	+1	0	0	No placebo was administered
7. Does the drug reach toxic concentrations in the blood or other body fluids?	+1	0	0	0	Unmonitored drug concentration
8. Did the adverse reaction worsen with increasing doses of the suspected drug or improve with reduced doses?	+1	0	0	0	No change in medication dosage
9. Has the patient ever been exposed to the same or similar drugs and experienced a similar reaction?	+1	0	0	0	Not previously exposed to drugs of the same type or class
10. Is there any objective evidence confirming this adverse reaction?	+1	0	0	1	The occurrence of pruritus and psoriasiform rash after medication confirms this reaction
Total score	-	-	-	7	

A total score of 9 or above indicates a definitive association between the pharmaceutical agent and the adverse reaction. A total score of 5-8 indicates a very likely association. A total score of 1-4 indicates a possible association. A total score of ≤0 indicates an association that is questionable or essentially non-existent. HCV, hepatitis C virus; NB-UVB, narrow-band ultraviolet B.

## Data Availability

The data generated in the present study may be requested from the corresponding author.
